# Outcome of acute myocardial infarction versus stable coronary artery disease patients treated with coronary bypass surgery

**DOI:** 10.1080/07853890.2020.1818118

**Published:** 2020-09-14

**Authors:** Markus Malmberg, Jarmo Gunn, Päivi Rautava, Jussi Sipilä, Ville Kytö

**Affiliations:** a Heart Center, Turku University Hospital and University of Turku, Turku, Finland; b Department of Public Health, University of Turku, Turku, Finland; c Turku Clinical Research Centre, Turku University Hospital, Turku, Finland; d Department of Neurology, North Karelia Central Hospital, Siun Sote, Joensuu, Finland; e Department of Neurology, University of Turku, Turku, Finland; f Center for Population Health Research, Turku University Hospital and University of Turku, Turku, Finland; g Administative Center, Hospital District of Southwest Finland, Turku, Finland

**Keywords:** Coronary artery disease, myocardial infarction, coronary artery bypass surgery, outcomes, Cohort study

## Abstract

**Objective:**

To study the long-term outcome differences between acute myocardial infarction (MI) and stable coronary artery disease (CAD) patients treated with coronary artery bypass grafting (CABG).

**Methods:**

We studied retrospectively patients with MI (*n* = 1882) or stable CAD (*n* = 13117) treated with isolated CABG between 2004 and 2014. Inverse propensity probability weight adjustment for baseline features was used. Median follow-up was 7.9 years.

**Results:**

In-hospital mortality (8.6% vs. 1.6%; OR 5.94; *p* < .0001) and re-sternotomy (5.5% vs. 2.7%; OR 2.07; *p* < .0001) were more common in MI patients compared to stable CAD patients. Hospital surviving MI patients had higher all-cause mortality (28.2% vs. 22.2%; HR 1.37; *p* = .002) and MACE rate (34.4% vs. 27.4%; HR 1.22; CI 1.00–1.50; *p* = .049) at 10-year follow-up. Cardiovascular mortality (15.9% vs. 12.7%; HR 1.36; *p* = .017) and rate of new myocardial infarction (12.0% vs. 9.8%; HR 1.40; *p* = .034) were also higher in MI patients during follow-up. In follow-up of stabilized first-year survivors, the difference in all-cause (26.5% vs. 20.7%; HR 1.40; *p* = .003) and cardiovascular (14.2% vs. 11.4%; HR 1.37; *p* = .027) mortality continued to increase between MI and stable CAD patients.

**Conclusion:**

MI patients have poorer short- and long-term outcomes compared to stable CAD patients after CABG and risk difference continues to increase with time.

**Key Messages**
1. Patients with myocardial infarction have poorer short- and long-term outcomes compared to stable coronary artery disease patients after coronary artery bypass grafting (CABG).
2. Higher risk of death continues also in stabilized first-year myocardial infarct survivors.
3. The importance of efficient secondary prevention and follow-up highlights in post-myocardial infarct population after CABG.

## 1. Introduction

Coronary artery disease (CAD) and its acute manifestation myocardial infarction (MI) are the leading causes of mortality globally and they significantly increase morbidity and the overall health care burden [1]. In acute MI and stable CAD, the choice of revascularization by coronary artery bypass grafting (CABG) or percutaneous coronary intervention (PCI) is based on clinical presentation, patients age and comorbidities and coronary lesion characteristics [2–4]. Revascularization by CABG improves outcomes in stable multivessel or left-main CAD [5] and has good results also in MI patients not suitable to percutaneous treatment [6,7]. Acute coronary syndrome (ACS), including both unstable angina pectoris (UA) and MI, is an independent risk-factor for early mortality after CABG [8,9]. Post-MI patients are known to be at high risk beyond acute phase after infarction [10]. Little is however known on long-term prognostic significance of MI for CABG-treated patients. Thus, we sought to investigate the short- and long-term outcome impact of undergoing CABG due to MI versus stable CAD in a real-world baseline adjusted population-based setting.

## 2. Materials and methods

### 2.1. Study design

We studied differences in outcomes of baseline adjusted MI and stable CAD patients treated with isolated CABG. Short-term outcomes were in-hospital all-cause mortality, re-sternotomy (for all operated patients) and duration of hospital stay (of hospital survivors). Sequential admissions in different wards and hospitals after surgery were all combined. Long-term outcomes were all-cause mortality, combined major adverse cardiovascular event (MACE; cardiovascular mortality, MI or stroke) and individual components of MACE. Long-term outcomes were studied in patients discharged alive after CABG. In addition, outcomes were studied in patients surviving without MACE for 1 year after CABG. Long-term outcomes were studied for 10-year occurrence after CABG. Follow-up ended for mortality 10 years after index hopitalization or 31.12.2016, whichever came first. For other outcome, follow-up ended 31.12.2014. Study design and outcomes are described in more detail in the Supplementary. The study was approved by the National Institute for Health and Welfare of Finland (permissions no: THL/143/5.05.00/2015 and THL/1569/5.05.00/2016), the Statistics Finland (TK53-1410-15) and the Social Insurance Institution of Finland (91/522/2015). Patient consent was waived due to study design.

### 2.2. Patient

All patients aged ≥18 who underwent CABG as primary operation between January 1, 2004 and December 31, 2014 were retrospectively recognized from the Care Register for Healthcare in Finland. This nationwide, mandatory registry includes data on all hospital admissions in Finland [11]. Myocardial infarction was recognized with ICD-10 code I21 as primary diagnosis and stable CAD with I25 as primary diagnosis. Bypass surgery for acute MI or stable CAD patients was performed in six public hospitals in Finland, which all were included in the study. Patients with prior cardiac surgery (including prior CABG), concomitant surgery of heart valves, surgery of aorta, surgery of other cardiac or pulmonary vasculature defects, bypass using other arterial grafts than *in situ* left or right internal thoracic artery, or any other coronary surgery were excluded. Co-morbidities were recognized from the Care Register for Healthcare and the Nationwide database of permissions for drug reimbursements in Finland using previously described ICD coding [12] and applicable drug purchase reimbursement codes (https://www.kela.fi/web/en/medicine-expenses). Each comorbidity was accounted separately in the adjustment for outcome analysis. Mortality data of patients was obtained from nationwide, mandatory cause of death registry held by Statistics Finland.

### 2.3. Statistical analysis

Inverse probability weighting (IPW) was used to balance baseline differences of MI and stable CAD patients [13]. Propensity score including age, sex, age*sex, atrial fibrillation, cerebrovascular disease, chronic pulmonary disease, dementia, diabetes, heart failure, hemi- or paraplegia, hypertension, liver disease, malignancy, peptic ulcer disease, peripheral vascular disease, prior MI, rheumatic disease, renal disease, type of by-pass graft, number of grafted anastomoses, study year and surgical centre were created using logistic regression. In order to improve balancing, propensity scores were trimmed to overlapping area. Inverse probability weights were calculated based on propensity score. Propensity scoring and IPW were re-performed for hospital survivors and first-year survivors. Weighting resulted in balanced groups (Table 1; Supplementary Table) with standardized difference of propensity score of 0.021 (variance ratio 1.01). Mean of unstabilized weights was 2.03 (min 1.01, max 81.2) and mean of stabilized weights was 1.00 (min 0.18, max 10.14).

**Table 1.** Features of myocardial infarction and stable coronary artery disease patients treated with coronary artery bypass grafting.

	Original cohort				Weighted cohorts		
Variable	All patients *n* = 15,059	Myocardial infarction *n* = 1882	Stable Coronary Artery Disease *n* = 13,177	SMD	Operated *n* = 15,059	Hospital survivors *n* = 14,740	First year survivors^a^ *n* = 13,429
					SMD	SMD	SMD
Age, years (SD)	67.1 (8.9)	67.7 (9.6)	67.0 (8.8)	0.07	0.02	0.02	0.03
Male sex	11,911 (79.1%)	1440 (76.5%)	10,471 (79.5%)	0.07	0.02	0.02	0.03
Co-morbidities							
Atrial fibrillation	1253 (8.3%)	138 (7.3%)	1115 (8.5%)	0.04	0.03	0.05	0.06
Cerebrovascular disease	835 (5.5%)	127 (6.8%)	708 (5.4%)	0.06	0.01	0.02	0.05
Chronic pulmonary disease	1162 (7.7%)	146 (7.8%)	1016 (7.7%)	0.01	0.03	0.03	0.05
Dementia	42 (0.3%)	8 (0.4%)	34 (0.3%)	0.03	0.00	0.01	0.01
Diabetes	3826 (25.4%)	502 (26.7%)	3324 (25.2%)	0.03	0.03	0.04	0.05
Heart failure	1702 (11.3%)	241 (12.8%)	1461 (11.1%)	0.05	0.01	0.03	0.03
Hemi- or paraplegia	8 (0.05%)	1 (0.05%)	7 (0.05%)	0.00	0.02	0.02	0.02
Hypertension	7640 (50.7%)	937 (49.8%)	6703 (50.9%)	0.02	0.04	0.05	0.03
Liver disease	47 (0.3%)	5 (0.3%)	42 (0.3%)	0.01	0.01	0.01	0.09
Malignancy	826 (5.5%)	124 (6.6%)	702 (5.3%)	0.05	0.05	0.06	0.06
Peptic ulcer disease	92 (0.6%)	21 (1.1%)	71 (0.5%)	0.06	0.04	0.04	0.05
Peripheral vascular disease	680 (4.5%)	101(5.4%)	579 (4.4%)	0.05	0.01	0.01	0.01
Prior myocardial infarction	3173 (21.1%)	542 (28.8%)	2631 (20.0%)	0.27	0.03	0.05	0.03
Rheumatic disease	561 (3.7%)	64 (3.4%)	497 (3.8%)	0.02	0.01	0.01	0.01
Renal disease	245 (1.6%)	35 (1.9%)	210 (1.6%)	0.02	0.03	0.01	0.03
Type of by-pass graft							
Only ITA	3215 (21.3%)	365 (19.6%)	2846 (21.6%)	0.05	0.02	0.02	0.01
Only venous	1066 (7.1%)	225 (12.0%)	841 (6.4%)	0.19	0.06	0.05	0.05
ITA and venous	10,778 (71.6%)	1288 (68.4%)	9490 (72.0%)	0.08	0.02	0.01	0.02
Number of grafted anastomoses							
1	3151 (20.9%)	370 (19.7%)	2781 (21.1%)	0.04	0.01	0.001	0.01
2	1956 (13.0%)	262 (13.9%)	1694 (12.9%)	0.03	0.03	0.02	0.01
3	4198 (27.9%)	542 (28.8%)	3656 (27.8%)	0.02	0.01	0.001	0.01
4	3675 (24.4%)	439 (23.3%)	3236 (24.6%)	0.03	0.002	0.01	0.01
5	1565 (10.6%)	202 (10.7%)	1393 (10.6%)	0.01	0.02	0.02	0.03
≥6	484 (3.2%)	67 (3.6%)	417 (3.2%)	0.02	0.02	0.02	0.04
Off pump surgery	204 (1.4%)	33 (1.8%)	171 (1.3%)	0.04	0.04	0.05	0.04
Operation year^b^				0.67	0.07	0.06	0.07
Operating Hospital (*n* = 6)				0.42	0.09	0.09	0.10

Differences between groups for all patients and for inverse probability weighted cohorts. ITA: internal thoracic arte; SMD: standardized mean difference (absolute value).

^a^Without major adverse cardiovascular event during first year after bypass surgery. Operated in 2004–2013.

^b^Operating years are presented in Supplementary Table.

Standardized difference scores were used for evaluation of effect sizes in baseline characteristics between groups. Differences between groups were studied Chi-Square test. Re-sternotomy and in-hospital mortality were studied using logistic regression. Mortality, MACE, MI and stroke were studied using the Kaplan–Meier method and Cox regression. Proportional hazard assumptions were confirmed by visual examination of Schoenfeld residuals. Cause-specific hazard models for competing risk due to death were applied in analysis of outcomes. Admission duration from surgery to discharge (beginning days) of hospital survivors was studied using negative binomial regression. Stable CAD was used as the reference in regression modelling. Adjustment for baseline covariates was performed by weighting with stabilized IPWs. Robust sandwich type estimates were used in regression analyses [14]. Median follow-up for mortality was evaluated by reverse Kaplan–Meier method. Results are given as the mean, median, percentage, hazard ratio (HR), relative risk (RR) or odds ratio (OR) with 95% confidence intervals (CI), interquartile range (IQR) or ± SD. A *P* value <0.05 was inferred statistically significant. Analyses were conducted using SAS version 9.4 (SAS Institute Inc., Cary, NC).

## 3. Results

Of all 15,059 patients 1882 (12.5%) were operated due to MI and 13,177 (87.5%) due to stable CAD. Proportion of MI patients increased significantly during study period from 3.0% in 2004 to 29.5% in 2014 (Table 1). Majority of all operated patients were men (79.1%). Women were slightly over represented in MI patients (23.5% females) compared to stable patients (20.5%; *p* = 0.003). Mean age of all patients was 67.1 (8.9) years (Table 1). Prior MI was more common in MI patients (Table 1). Revascularization using only venous grafts was more common in patients with MI, but the number of grafted anastomoses did not differ between study groups (Table 1). Median time from admission to the surgery in MI group was 2 days (IQR 1–6 days). Median follow-up was 7.9 years.

### 3.1. In-hospital outcomes

In-hospital outcomes after CABG were poorer in patients with MI (Table 2). Adjusted in-hospital mortality was 8.6% with MI and 1.6% with stable CAD patients (OR 5.94; CI 4.44–7.94; *p* < .0001). Re-sternotomy after CABG was performed to 5.5% of MI patients and 2.7% of stable CAD patients (OR 2.07; CI 1.46–2.94; *p* < .0001). Duration of hospital admission from operation to discharge was longer in MI patients (median 13 days, IQR 10-118) than in stable CAD patients (median 11, IQR 9–14; adjusted *p* < .0001) with estimated RR per 1 day increase in admission of 1.28 (CI 1.18–1.39; *p* < .0001).

**Table 2.** Results of unadjusted and stabilized inversed weight (IPW) adjusted outcomes analyses.

		Myocardial infarction vs. stable CAD patients			
Outcome after CABG	Estimator	Unadjusted (95% CI)	*p* Value	IPW adjusted (95% CI)	*p* Value
In-hospital outcomes					
All-cause mortality	OR	4.63 (3.67–5.83)	<.0001	5.94 (4.44–7.94)	<.0001
Re-sternotomy	OR	1.57 (1.23–2.01)	.0003	2.07 (1.46–2.94)	<.0001
Admission duration (per 1 day)	RR	1.17 (1.13–1.21)	<.0001	1.28 (1.18–1.39)	<.0001
Hospital survivors: 1-year outcomes					
All-cause mortality	HR	1.46 (1.08–1.96)	.013	1.33 (0.83–2.11)	.234
MACE	HR	2.22 (1.82–2.71)	<.0001	1.60 (1.16–2.21)	.004
Cardiovascular mortality	HR	1.50 (1.06–2.10)	.020	1.34 (0.79–2.27)	.279
Myocardial infarction	HR	3.89 (2.92–5.17)	<.0001	2.33 (1.49–3.64)	.0002
Stroke	HR	1.53 (1.08–2.17)	.017	1.42 (0.80–2.52)	.226
Hospital survivors: 10-year outcomes					
All-cause mortality	HR	1.16 (1.02–1.33)	.027	1.38 (1.12–1.68)	.002
MACE	HR	1.50 (1.32–1.71)	<.0001	1.22 (1.00–1.50)	.049
Cardiovascular mortality	HR	1.30 (1.10–1.54)	.002	1.37 (1.06–1.76)	.015
Myocardial infarction	HR	2.03 (1.66–2.47)	<.0001	1.40 (1.03–1.91)	.034
Stroke	HR	1.29 (1.04–1.60)	.022	0.93 (0.65–1.32)	.676
First-year survivors: 10-year outcomes					
All-cause mortality	HR	1.14 (0.98–1.32)	.091	1.40 (1.12–1.73)	.003
MACE	HR	1.26 (1.04–1.45)	.018	1.09 (0.86–1.40)	.474
Cardiovascular mortality	HR	1.28 (1.05–1.56)	.013	1.37 (1.04–1.81)	.027
Myocardial infarction	HR	1.22 (0.91–1.65)	.181	1.12 (0.75–1.69)	.585
Stroke	HR	1.18 (0.90–1.55)	.227	0.77 (0.51–1.15)	.197

CABG: coronary artery bypass grafting; CAD: coronary artery disease; CI: confidence interval; HR: hazard ratio; MACE: major adverse cardiovascular event; OR: odds ratio; RR: rate ratio.

### 3.2. First year outcomes

**Figure 1.**
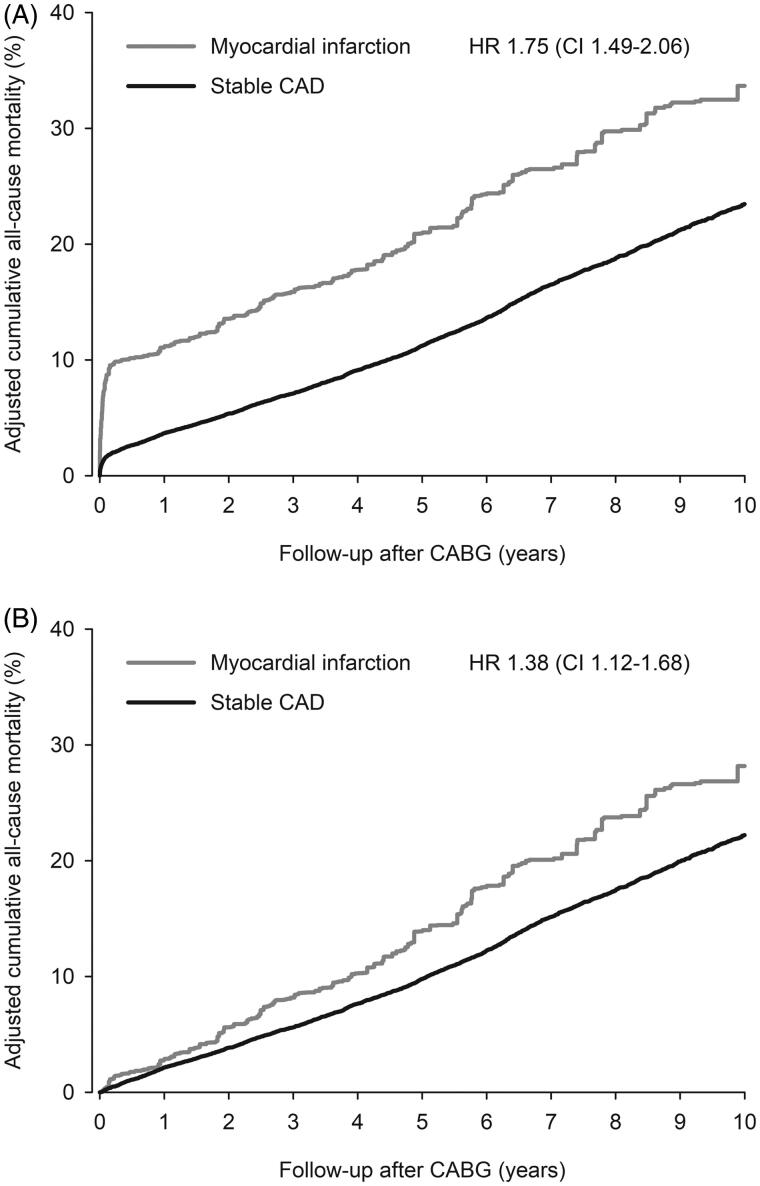
Cumulative adjusted all-cause mortality of all (A) and hospital surviving (B) myocardial infarction (MI) and stable coronary artery disease (CAD) patients after coronary artery bypass grafting (CABG). HR: inverse probability weight adjusted hazard ration. CI = 95% confidence interval.

**Figure 2.**
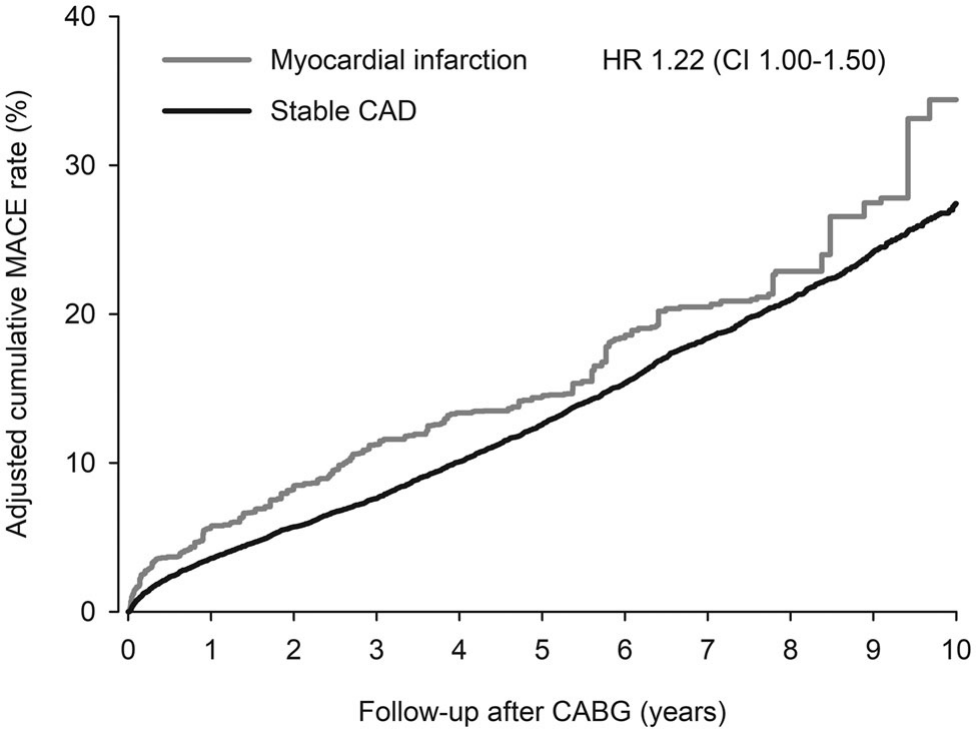
Cumulative adjusted occurrence of major adverse cardiovascular events (MACE) on hospital surviving myocardial infarction (MI) and stable coronary artery disease (CAD) patients after coronary artery bypass grafting (CABG). HR: inverse probability weight adjusted hazard ration. CI = 95% confidence interval.

**Figure 3.**
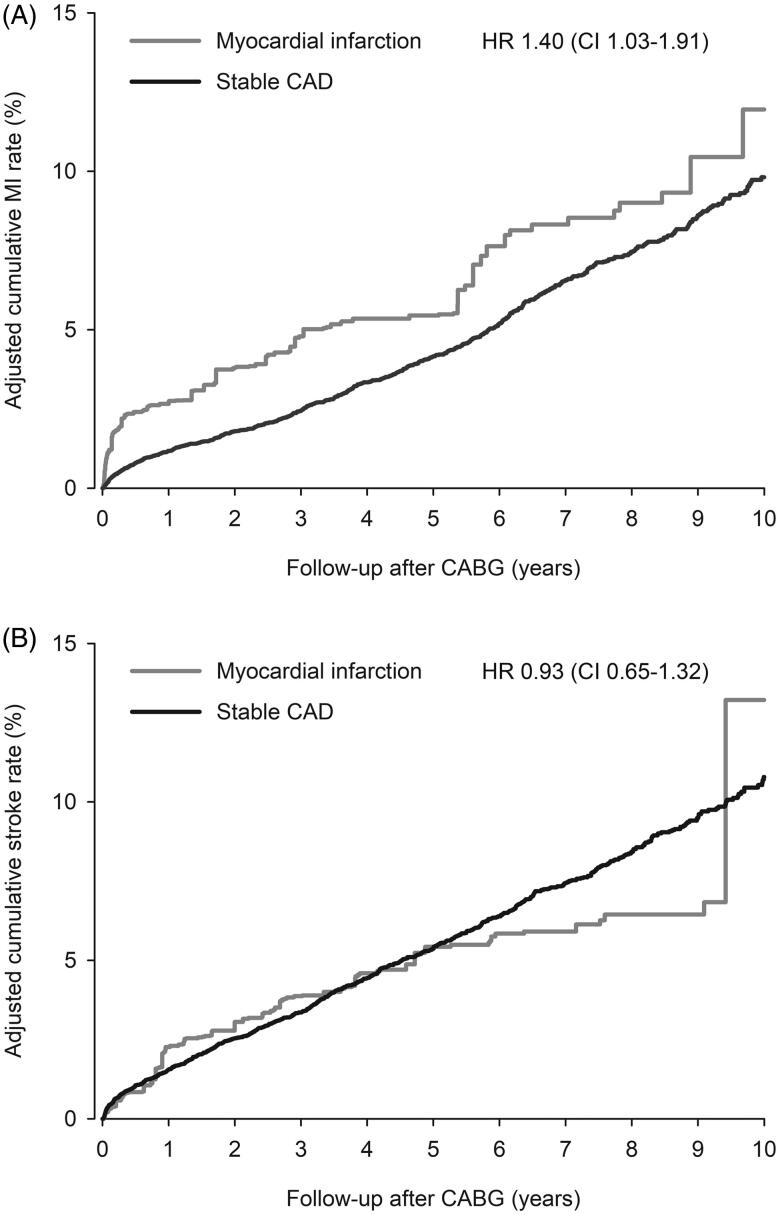
Cumulative adjusted occurrence of myocardial infarction (A) and stroke (B) in hospital surviving myocardial infarction (MI) and stable coronary artery disease (CAD) patients after coronary artery bypass grafting (CABG). HR: inverse probability weight adjusted hazard ration. CI = 95% confidence interval.

All-cause mortality at 1-year among hospital survivors after CABG was 2.8% in MI group and 2.1% in stable CAD group (HR 1.33; CI 0.83–2.11; *p* = .23, Figure 1). Cumulative adjusted MACE rate of hospital survivors at 1-year was 5.7% in MI and 3.6% in stable CAD patients (HR 1.60; CI 1.16–2.21; *p* = .004, Figure 2). First-year adjusted all-cause mortality of all operated patients was 11.2% in MI and 3.7% in stable CAD groups (HR 3.20; CI 2.53–4.04; *p* < .0001, Figure 1). One-year cardiovascular mortality rate in hospital survivors was 2.1% in MI vs. 1.6% in stable CAD group (HR 1.34; CI 0.79–2.27; *p* = .279). Myocardial infarction occurred to 2.7% of MI and 1.2% of stable CAD patients (hospital survivors) within first year after CABG (HR 2.33; CI 1.49–3.64; *p* = .0002, Figure 3). Stroke rate was 2.3% in MI and 1.6% in stable CAD patients at 1-year (HR 1.42; CI 0.80–2.532; *p* = .226, Figure 3).

### 3.3. Ten-year outcomes

All-cause mortality of hospital survivors was 28.2% in MI and 22.2% (HR 1.38; CI 1.12-1.68; *p* = .002, Figure 1). Among all operated patients the 10-year all-cause mortality was 33.7% in MI and 23.5% in stable CAD (HR 1.75; CI 1.49–2.06; *p* < .0001, Figure 1). Ten-year MACE rate was 34.4% in MI patients compared to 27.4% in stable CAD patients (HR 1.22; CI 1.00–1.50; *p* = .049, Figure 2). Cumulative incidence of cardiovascular mortality at 10 years was 15.9% in MI group and 12.7% in stable CAD group in hospital survivors (HR 1.36; CI 1.06–1.75; *p* = .017). Myocardial infarction rate within 10-years was 12.0% in MI group and 9.8% in stable CAD group (HR 1.40; CI 1.03–1.90; *p* = .034, Figure 3). Occurrence of stroke did not differ significantly within 10-year follow-up (13.2% in MI vs. 10.8% in stable CAD; HR 0.93; CI 0.65–1.32; *p* = .676, Figure 3).

### 3.4. Outcomes of first-year survivors

Difference between MI and stable CAD patients in all-cause (26.5 vs. 20.7%; HR 1.40; CI 1.12–1.73; *p* = .003) and cardiovascular mortality (14.2 vs. 11.4%; HR 1.37; CI 1.04–1.81 *p* = .027) continued to increase during 9-year follow-up beyond the first year after CABG. Rates of new MACE (30.5 vs. 25.1%), MI (9.9 vs. 8.8%), or stroke (11.3 vs. 9.5%) were comparable in follow-up beyond first year after CABG (Table 2.)

## 4. Discussion

This population-based, multicenter nationwide study investigated the outcome differences of baseline adjusted patients with MI or stable CAD who underwent CABG. Myocardial infarction patients had poorer outcomes at short-term, but also at long-term follow-up. Importantly, stabilized post-MI survivors continued to be at higher risk of mortality in long-term follow-up.

In agreement with previous studies of ACS and MI patients [9,15], we found in-hospital mortality to be significantly higher when CABG was performed for patients with MI (8.6%) compared to patients with stable CAD (1.6%). This mortality rate is well in line with previous findings of 2.7–21.6% early mortality after CABG in MI patients [7,15]. Increased mortality of MI patients is associated with higher risk of both procedural (e.g. early bypass dysfunction) and MI related complications [7,15], reflected also to a longer hospitalization period after surgery found in our data. Optimal timing of CABG after MI for maximal benefit-risk gain is unclear [15,16]. Urgent revascularization may be necessary, but night-time emergency surgery with reduced operating team may also contribute for poorer outcome in MI [15,16]. Our data does not allow studying the timing of surgery after MI, but previous results suggests that optimal of timing for CABG can be appropriately determined by clinicians [17].

We also found that the rate of re-sternotomy reflecting major bleeding to be significantly higher after CABG for MI patients compared to stable CAD patients (5.5 vs. 2.7%). Due to increased platelet action, it is a common practice advocated by guidelines [18] to pretreat MI patients with antiplatelet adenosine diphosphate P2Y12 receptor antagonist (clopidogrel, prasugrel or ticagrelor) before coronary angiography. However, in recent findings, the increased re-sternotomy and bleeding rates [19] in MI patients undergoing CABG and non-effectiveness of pre-treatment prior to PCI [20], question the benefits of routine P2Y12 inhibitor preloading in NSTEMI patients, especially if left main or multivessel disease is suspected.

Worse prognosis of MI patients after surgery continued beyond hospital discharge. Occurrence of MACE was high in post-CABG MI patients with 34% of hospital surviving MI patients facing cardiovascular death, new MI or stroke within 10 years. Difference compared to stable CAD patients (MACE rate 27.4%) was driven by higher rates of new onset MI and cardiovascular mortality. Previous data on long-term outcomes of CABG-treated MI patients is sparse. Japanese single-centre study of 1233 patients found comparable late mortality and major adverse cardiac and cerebrovascular events between propensity matched ACS and stable CAD patients [9]. However, patients with MI have poorer prognosis than those with UA who are also included in ACS [21]. Despite the fact that patients who underwent CABG following unstable angina had better long-term survival than patients with NSTEMI, patients with unstable angina remain at high risk also after PCI when compared with stable CAD [22,23]. Although long-term mortality and cardiovascular outcomes are more common in MI patients after CABG, quality of life 10 years after surgery is nevertheless found to be excellent and comparable to matched control population in both acute and stable CAD post-CABG patients [24].

Importantly, we found the stabilized post-MI patients to have poorer survival also beyond first MACE-free year after CABG. This continuing risk-difference compared to non-MI CAD patients is most likely associated with differences in conventional CAD risk factors and MI-related myocardial injury, but also to other factors such as coronary plaque stability [25] and thrombogenesis [26]. Detailed mechanisms remain to be further clarified. There is substantial evidence on efficient pharmacotherapy including, e.g. beta-blockers, angiotensin-converting enzyme inhibitors, antiotensin-II receptor blockers, statins, antiplatelet and anticoagulative agents in addition to non-pharmacotherapeutic intervention including, e.g. smoking cessation, rehabilitation and physical exercise in preventing cardiovascular outcomes in CAD and post-MI patients [27]. Although there is evidence of improving usage of secondary prevention [28], improvements are still called upon for initiation and adherence of effective therapies [29]. Our finding underlines the importance of usage of effective secondary prevention therapy especially in post-MI population after CABG. Improving long-term adherence to effective secondary prevention requires adequate follow-up program especially in post-MI CABG patients.

There are limitations in this study. The major limitation is retrospective design with usage of registry data. In this study, combination of previously validated nationwide, mandatory by law registries was used [30]. Treating physicians were responsible for diagnoses in the registries and coding errors are therefore possible. We did not have access to detailed clinical or operative information **(**e.g. SYNTAX or EuroScore), or to usage of pharmacotherapies. Although registries used are mandatory and have good coverage, it is possible that some co-morbidities and non-fatal outcomes may be underestimated. Co-morbidity burden in different studies varies due to differences in patient populations, data collection and in definitions. In our study, the rates of major co-morbidities (e.g. diabetes, prior MI, heart failure, peripheral vascular disease) were similar to those reported in prospective study of ACS patients treated with CABG [9] or large-scale Swedish registry study of CABG patients [31]. Proportions of atrial fibrillation and hypertension patients have larger variation between studies [9,31]. It is however not likely that these limitations would have significantly different influence on study groups in the current study setting. Propensity score weighting for number of baseline features was used to balance study groups, but it is nevertheless possible that additional non-recognizable co-founders may impact the results.

Since we did not study post-CABG parameters (such as pharmacotherapy, laboratory results including markers of myocardial cell destruction such as creatine kinase, or development of left ventricular systolic function after MI), our study is unable to highlight detailed mechanisms of poorer outcome in MI patients. It is plausible that reduced left ventricle ejection fraction contributes for poorer long-term outcome in CABG patient following MI [7].

As a conclusion, MI patients have poorer short- and long-term outcomes compared to stable CAD patients after CABG. Higher risk of death continues also in stabilized first-year MI survivors. These results underline the importance of efficient secondary prevention and follow-up in post-MI population after CABG.

## Supplementary Material

Supplemental Material
