# Supplementary material for: Outcome of acute myocardial infarction versus stable coronary artery disease patients treated with coronary bypass surgery
Source: Ann Med. 2020 Sep 14;53(1):70–7. doi: 10.1080/07853890.2020.1818118 (PMC7877950; doi:10.1080/07853890.2020.1818118)
Supplement: Supplemental Material [file IANN_A_1818118_SM6243.docx]

Supplement Methods

Data sources

Following obligatory, nationwide data used for patient and outcome identification were collected:

- Data of all hospital admissions to medical, surgical, neurological, neurosurgical or intensive care units in Finland including discharge diagnoses (International Classification of Diagnosis / ICD-10) and operational codes (Nordic Classification of Surgical Procedures) from the Care Register for Healthcare in Finland (CRHF) held by the National Institute for Health and Welfare of Finland.
- Data of all drug purchase reimbursement permissions and applicable ICD-codes held by the Social Insurance Institution of Finland.
- Mortality data including date and causes of death held by the Statistics Finland.

Inclusion criteria

- Age ≥ 18 years.
- Coronary artery by-bass grafting surgery (CABG).
- Stable coronary artery disease:
  - Coronary artery disease: ICD-10 code I25.* (primary diagnosis)
- Myocardial infarction (primary diagnosis): ICD-10 code I21*
  - - STEMI: ICD-10 codes I21.0*, I21.1*, I21.3*.
    - NSTEMI: ICD-10 codes I21.4* or I21.9*.

Exclusion criteria

- Prior cardiac surgery (including prior CABG).
- Concomitant surgery of heart valves, aorta, or other cardiac or pulmonary vasculature defects.
- Bypass using free or gastroepiploic arterial grafts, or other coronary surgery.
- From stable coronary artery disease group
  - Acute coronary syndrome
  - Urgent/emergency revascularization

Outcomes

*Short-term*

- All-cause mortality.
- Re-sternotomy during surgical admission.
- Duration admission from CABG to discharge (beginning days, hospital survivors).

*Long-term*

- Major adverse cardiovascular event (MACE): Cardiovascular death, myocardial infarction, or stroke.
- Cardiovascular mortality: Underlying cause of death: ICD-10 code I*.
- Myocardial infarction: ICD-10 codes I21*and I22*
- Stroke: ICD-10 codes I60*-I64*.
- All-cause mortality.
- New MI and stroke included both admissions with MI / stroke as the primary cause of admission and death with MI/stroke as any cause of death.

Follow-up

- Follow-up time 10 years.
- All-cause and cardiovascular mortality data available up to Dec 31^st^ 2016.
- MACE, MI, and stroke data available up to Dec 31^st^ 2014.

| Operation year | All patients n=15059 | Myocardial infarction  n=1882 | Stable Coronary Artery Disease  n=13177 |
| --- | --- | --- | --- |
| 2004 | 1658 (11.0%) | 50 (3.0%) | 1608 (97.0%) |
| 2005 | 1642 (10.9%) | 85 (5.2%) | 1557 (94.8%) |
| 2006 | 1388 (9.2%) | 109 (7.9%) | 1279 (92.1%) |
| 2007 | 1357 (9.0%) | 118 (8.7%) | 1239 (91.3%) |
| 2008 | 1584 (10.5%) | 137 (8.7%) | 1447 (91.4%) |
| 2009 | 1344 (8.9%) | 170 (12.7%) | 1174 (87.4%) |
| 2010 | 1291 (8.6%) | 220 (17.0%) | 1071 (83.0%) |
| 2011 | 1323 (8.8%) | 206 (15.6%) | 1117 (84.4%) |
| 2012 | 1305 (8.7%) | 235 (18.0%) | 1070 (82.0%) |
| 2013 | 1149 (7.6%) | 252 (21.9%) | 897 (78.1%) |
| 2014 | 1018 (6.8%) | 300 (29.5%) | 718 (70.5%) |

Supplement Table. Year of coronary atery bypass surgery in study population. Percentage for all study patients is given from all surgeries during study perios. Percentages of myocardial infarction and stable coronary artery disease are given per individual year.
